# A genotype-to-phenotype approach suggests under-reporting of single nucleotide variants in nephrocystin-1 (*NPHP1*) related disease (UK 100,000 Genomes Project)

**DOI:** 10.1038/s41598-023-32169-4

**Published:** 2023-06-09

**Authors:** Gary Leggatt, Guo Cheng, Sumit Narain, Luis Briseño-Roa, Jean-Philippe Annereau, The Genomics England Research Consortium, J. C. Ambrose, P. Arumugam, R. Bevers, M. Bleda, F. Boardman-Pretty, C. R. Boustred, H. Brittain, M. A. Brown, M. J. Caulfield, G. C. Chan, A. Giess, J. N. Griffin, A. Hamblin, S. Henderson, T. J. P. Hubbard, R. Jackson, L. J. Jones, D. Kasperaviciute, M. Kayikci, A. Kousathanas, L. Lahnstein, A. Lakey, S. E. A. Leigh, I. U. S. Leong, F. J. Lopez, F. Maleady-Crowe, M. McEntagart, F. Minneci, J. Mitchell, L. Moutsianas, M. Mueller, N. Murugaesu, A. C. Need, P. O‘Donovan, C. A. Odhams, C. Patch, D. Perez-Gil, M. B. Pereira, J. Pullinger, T. Rahim, A. Rendon, T. Rogers, K. Savage, K. Sawant, R. H. Scott, A. Siddiq, A. Sieghart, S. C. Smith, A. Sosinsky, A. Stuckey, M. Tanguy, A. L. Taylor Tavares, E. R. A. Thomas, S. R. Thompson, A. Tucci, M. J. Welland, E. Williams, K. Witkowska, S. M. Wood, M. Zarowiecki, Christine Gast, Rodney D. Gilbert, Sarah Ennis

**Affiliations:** 1 University of Southampton, Duthie Building (MP 808), Southampton General Hospital, Tremona Road Shirley, Southampton, SO16 6YD UK; 2 Medetia, Imagine Institute for Genetic Diseases, 24 Boulevard du Montparnasse, 75015 Paris, France; 3 Wessex Kidney Centre, Portsmouth Hospitals University NHS Trust, Southwick Hill Road, Cosham, Portsmouth, PO6 3LY UK; 4 Southampton Children’s Hospital, Southampton General Hospital, Tremona Road Shirley, Southampton, SO16 6YD UK; 5 University Hospital Southampton NHS Foundation Trust, Southampton General Hospital, Tremona Road Shirley, Southampton, SO16 6YD UK; 6 Genomics England, London, UK; 7 William Harvey Research Institute, Queen Mary University of London, London, EC1M 6BQ UK; 8 University of East Anglia, Norwich, UK

**Keywords:** Medical genetics, Molecular medicine, Nephrology, Disease genetics

## Abstract

Autosomal recessive whole gene deletions of nephrocystin-1 (*NPHP1*) result in abnormal structure and function of the primary cilia. These deletions can result in a tubulointerstitial kidney disease known as nephronophthisis and retinal (Senior–Løken syndrome) and neurological (Joubert syndrome) diseases. Nephronophthisis is a common cause of end-stage kidney disease (ESKD) in children and up to 1% of adult onset ESKD. Single nucleotide variants (SNVs) and small insertions and deletions (Indels) have been less well characterised. We used a gene pathogenicity scoring system (GenePy) and a genotype-to-phenotype approach on individuals recruited to the UK Genomics England (GEL) 100,000 Genomes Project (100kGP) (n = 78,050). This approach identified all participants with *NPHP1*-related diseases reported by NHS Genomics Medical Centres and an additional eight participants. Extreme *NPHP1* gene scores, often underpinned by clear recessive inheritance, were observed in patients from diverse recruitment categories, including cancer, suggesting the possibility of a more widespread disease than previously appreciated. In total, ten participants had homozygous CNV deletions with eight homozygous or compound heterozygous with SNVs. Our data also reveals strong in-silico evidence that approximately 44% of *NPHP1* related disease may be due to SNVs with AlphaFold structural modelling evidence for a significant impact on protein structure. This study suggests historical under-reporting of SNVS in *NPHP1* related diseases compared with CNVs.

## Introduction

Nephrocystin-1 (*NPHP1*) was first localised to chromosome 2q13 before a sizeable homozygous deletion was found in this same region^1^. Saunier et al. and Hildebrandt et al. identified *NPHP1* within the minimum deletion interval along with another gene, *MALL*^2,3^. They both noted additional single nucleotide variants (SNVs) in *NPHP1* but not in *MALL* in patients with a heterozygous deletion. Saunier et al*.* later demonstrated that the *NPHP1* gene was flanked by two inverted 358 kilobase (kb) low copy repeats (LCRs) and that these both included a smaller LCR of 45 kb, containing the patient's deletion breakpoints^4^.

Human chromosome two has arisen from the fusion of two ancestral great ape chromosomes, and the telomere-telomere fusion point is located at 2q13. Telomeres have several repeat sequences, which may explain the presence of these LCRs^5^. LCRs are found in 5–15% of the human genome and provide a point where nonallelic homologous recombination (NAHR) mediated cross-over can occur^5,6^. This unequal cross-over results in structural variation, including copy number variants (CNVs) such as duplications, deletions or inversions^5^.

*NPHP1* is localised to the connecting cilia in photoreceptor cells and to the base of the primary renal cilium (known as the transition zone) of renal epithelial cells of the collecting ducts^7,8^. Primary cilia are near-ubiquitous sensory organelles that protrude from the cell surface^9^. Diseases associated with *NPHP1* are therefore known as ciliopathies^10^.

Homozygous deletion of *NPHP1* is the commonest cause of nephronophthisis (20–25% of cases)^11–13^. Nephronophthisis, which means 'vanishing nephron' in Greek, is a tubulointerstitial kidney disease classically divided into three categories, based on the age of presentation: infantile, juvenile, and, less commonly, adolescent^14^. Ultrasound examination reveals small to normal-sized kidneys, with increased echogenicity due to fibrosis and loss of corticomedullary differentiation. Small corticomedullary cysts may develop later in the disease course due to loss of normal tissue^14^. The infantile form differs most significantly from the other forms with *in-utero* onset, oligohydramnios and ESKD in the first two years of life but with enlarged, rather than normal or shrunken kidneys and more widespread cyst development^15^. Juvenile and adolescent nephronophthisis may be associated with polyuria and polydipsia preceding the development of chronic kidney disease as well as growth retardation^14^.

In addition to nephronophthisis, 15% of patients have retinal dystrophy (Senior–Løken syndrome) or neurological (Joubert syndrome) phenotypes^16^. The retinal phenotype may be mild^17^. Joubert syndrome (JS) is characterised by cerebellar ataxia, mental retardation, hypotonia, and neonatal respiratory dysregulation^18^. The cerebellar malformation can be seen on MRI scans as the so-called 'molar tooth sign' (MTS). The malformations are less severe in patients with *NPHP1* mutations with milder JS than with other gene causes^18–20^.

The homozygous deletion of *NPHP1* is fully penetrant and, therefore, pathognomonic of the nephronophthisis phenotype^11,13^. It is well established that nephronophthisis is a leading cause of ESKD in children^14^. However, more recent data from Snoek et al*.* identified homozygous deletions in *NPHP1* in 0.9% of adult onset ESKD^21^. One individual first reached ESKD as late as 61 years old. 69% of patients had a prior diagnosis other than nephronophthisis, tubulointerstitial or cystic kidney disease^21^. Although reported in association with nephronophthisis, small insertions and deletions (indels) and single nucleotide variants (SNVs) are less well characterised in *NPHP1*^22^.

While single variant association testing has successfully identified common disease variants, it remains underpowered for rare variants even with high case numbers^23^. We, therefore, applied our gene-level scoring system, GenePy^24^. GenePy is a software that transforms variant level data into gene-level data. GenePy generates a score of whole gene pathogenicity for each person by incorporating in silico deleteriousness metrics, population allele frequency and observed zygosity for each variant^24^. The effect of multiple variants within a gene is combined into a single cumulative gene pathogenicity score for each individual. GenePy scores are continuous but do not follow a normal distribution. Higher GenePy scores are intuitive at a base level as higher scores represent greater pathogenic mutational burden due to rare and deleterious mutations. GenePy scores can be used as the basis to prioritise large numbers of candidate variants.

The UK Genomics England (GEL) 100,000 Genomes Project (100kGP), which completed recruitment in 2018, aimed to sequence the genomes of patients with cancer and rare disease and linked this data with digital clinical records. The aims were to improve clinical diagnosis, tailor therapies, enable new scientific discoveries and link research with a clinically integrated genomic medicine service in the UK National Health Service (NHS)^25^.

Using whole-genome sequencing and longitudinal clinical data from the 100kGP project; we aimed to identify pathogenic variation in *NPHP1* and associated phenotypes using a genotype-to-phenotype approach. We generated GenePy scores for *NPHP1* for all individuals recruited to the 100kGP and further incorporated copy number variation (CNV) data. We also interrogated the frequency of CNVs and SNVs in individuals recruited to 100kGP and assessed the evidence for SNVs contributing to the phenotype. We applied a gene first approach or genotype-to-phenotype approach by considering the genotypes of all individuals recruited to the 100kGP irrespective of their disease. We then undertook reverse phenotyping to relate the phenotypic spectrum associated with differing mutations.

## Methods

### Ethical

The 100,000 genomes project was approved by the National Research Ethics Service Research Ethics Committee for East of England—Cambridge South Research Ethics Committee. All methods were carried out in accordance with relevant guidelines and regulations. Informed consent was obtained from all subjects and/or their legal guardian(s) as part of the original study.

### Recruitment

Access to the GEL dataset for the project (research registry ID 109) was approved by the renal GEL clinical interpretation partnership (GeCIP), and all analyses were conducted within the GEL research environment. Patients with GEL-defined eligibility criteria for either rare disease or cancer consented to germline whole-genome sequencing linked to clinical phenotype data, including longitudinal electronic health records. Specific Human Phenotype Ontology (HPO) data were collected at the time of recruitment depending on the participant's recruitment category^26^. Additional longitudinal phenotype data included the hospital episode statistics (HES) database containing details of all admission, emergency and outpatient appointments at NHS hospitals in England recorded as ICD-10 codes^27^. The majority of participants provided blood samples, although a small number provided saliva for germline DNA extraction. In addition to germline DNA, some participants recruited to the cancer arm also provided somatic tumour cell DNA.

### Phenotype data

Phenotype data was extracted from LabKey Main programme interim data release v11.0^28^. Human Phenotype Ontology (HPO) terms were provided by recruiting Genomic Medicine Centres (GMC) according to the GEL Protocol^29^.

### Computational pipeline

Genomic data was sourced from an aggregate multi-sample variant call format (VCF; aggV2) main release version 11 (17/12/20). All samples were sequenced with 150 bp paired-end reads in a single lane of an Illumina HiSeq X instrument. Raw sequencing output in the form of a binary base call (BCL) file was processed on the Illumina North Star Version 4 Whole Genome Sequencing Workflow (NSV4, v. 2.6.53.23), which comprises the iSAAC Aligner (v. 03.16.02.19) and Starling Small Variant Caller (v.2.4.7). CNVs were called by the Genomics England pipeline using Illumina canvas software^30^. Samples were aligned to the NCBI Genome Reference Consortium Human Build 38 assembly with decoy sequences. Single sample gVCFs were aggregated using Illumina gVCF genotyper (v.2019.02.26).

A multisource gVCF was extracted from the aggregated gVCF from which the *NPHP1* gene locus (defined using NCBI GRCh38/hg38 (chr2:110,123,335–110,205,062)) was selected using bcftools 1.6^31,32^.

Variants meeting minimum quality standards (GQ > 20, mean GQ of > 35, DP > 10 and maximum missingness of 70%) were identified using vcftools 0.1.16 and retained for downstream analysis (n = 12,780)^33^.

### GenePy

In order to generate whole gene pathogenicity scores, we applied the GenePy algorithm v.1.3 for both coding and non-coding variants^24,34^. The GenePy score (S) for a given gene (g) and individual (h) is the sum of the effect of all variants (k) where each biallelic variant locus (i) in a gene is weighted according to its predicted deleteriousness (using CADD(Di), zygosity and global allele frequency (gnomAD v3.0)) (f).

$$S_{gh} = - \sum\limits_{i = 1}^{k} {D_{i} \log_{10} (f_{i1} \cdot f_{i2} )}$$

The VCF was then annotated using ANNOVAR 1.0 for gnomAD_version 3.0 allele frequency data (f) and the RefGene database of genes^35^. Combined Annotation Dependent Depletion (CADD) v1.6 was used to annotate for deleteriousness (D)^36^. We limited variants to those with CADD PHRED scores greater than 15 (n = 214). This cut-off was chosen as it represents the median value for all possible canonical splice site changes and non-synonymous variants in CADD.

### Copy number variation (CNV)

The GenePy algorithm is streamlined to include indels and SNVs represented in VCF files but does not systematically incorporate CNV data. Therefore, a GenePy score for CNV whole gene deletions of *NPHP1* was integrated with the whole gene score by arbitrarily assigning (D) as the maximal CADD 1.6 score observed for a stop-gain variant within the *NPHP1* gene and allele frequency using gnomAD structural variant v2 (f = 1.798 × 10^–3^).

### Structural modelling

The mutations identified were modelled both in the Missense 3D Database and using Maestro Suite v13.1 and BioLuminate 4.6 Release 2022-1 (Maestro & BioLuminate, Schrödinger, LLC, New York, NY, 2021)^37–41^. Mutations were identified to be pathogenic following the criteria: (1) the substitution replaces a buried charged residue with an uncharged residue, (2) substitution disrupts all side-chain/side-chain H-bond(s) and/or side-chain/main-chain bond(s) H-bonds, (3) substitution breaks all side-chain/side-chain H-bond(s) and/or side-chain/main-chain H-bond(s) formed by the wild type which was buried, (4) substitution leads to an expansion or contraction of the cavity volume of ≥ 70 Å^3^, (5) the substitution breaks a salt bridge formed by wild type which was buried. The maximum N–O bond length is 5.0 Å.

## Results

**Figure 1:**
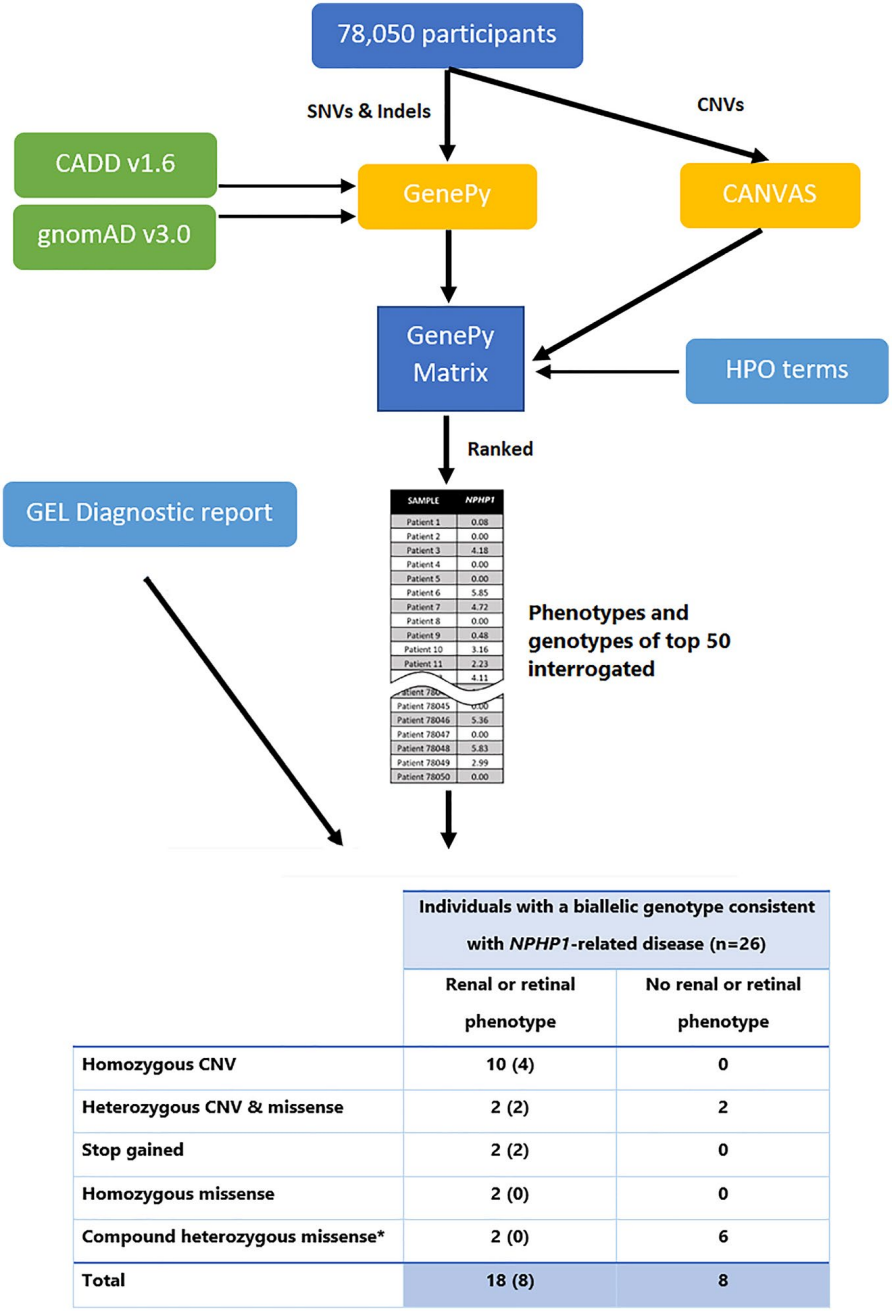
Bioinformatic workflow with numbers of participants with biallelic genotypes for monogenic *NPHP1*-related disease. Comparison of genotype-to-phenotype approach (participants with and without penetrant renal or retinal phenotypes) and those reported by the GEL clinical pipeline are in brackets. This approach was fully sensitive to pick up individuals as solved by the GEL clinical interpretation pipeline at the time of writing.

The genotype-to-phenotype approach incorporating SNVs, indels and CNVs into ranked *NPHP1* GenePy scores identified renal, retinal, or neurological phenotypes amongst the top-ranked individuals (see Table 1). Age at onset of disease is likely to be overestimated, as HES data was only available from the date of recruitment onwards. In total 26 participants were identified with biallelic recessive genotypes consistent with monogenic *NPHP1*-related disease. Our approach identified eighteen patients with a renal or retinal phenotype and a genotype constant with the monogenic autosomal recessive disease. The NHS genomic medicine centres have previously reported eight of these. In total ten homozygous CNV whole-gene deletions of *NPHP1* were discovered. Homozygous or compound heterozygous SNVs consistent with monogenic disease were identified in eight patients with renal, retinal, or neurological phenotypes (see Fig. 1).

**Table 1:**
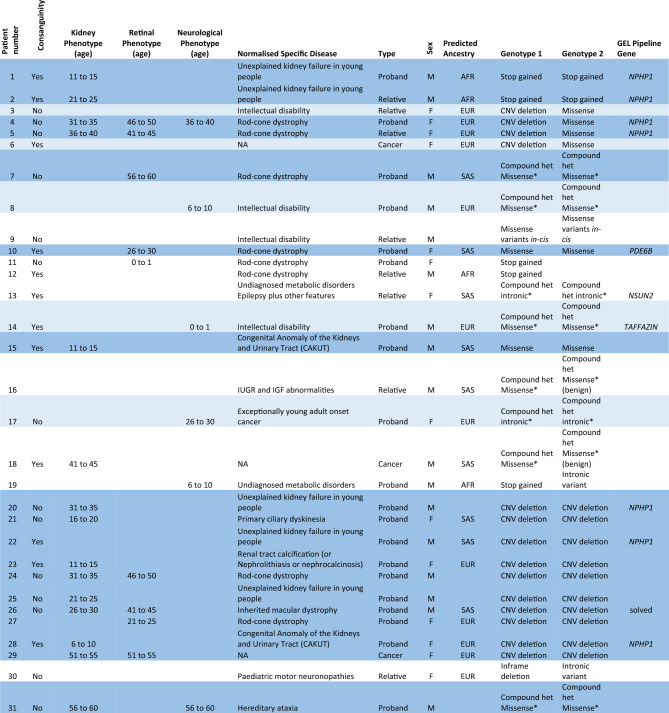

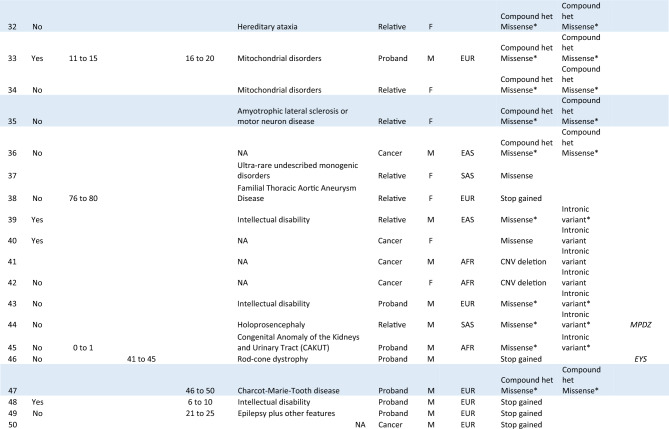
Top 50 GenePy scores from 78,050 participants across the Genomics England recruitments, including cancer and rare disease.

Individuals in dark blue (n = 18) have both a genotype and phenotype consistent with *NPHP1* related disease. Participants highlighted in light blue (n = 8) have a genotype consistent with *NPHP1* related disease but no phenotype recorded in the Genomics England research environment. This may either be because of inaccurate phenotyping, documentation or because they are yet to present to a healthcare professional or because of incompletely penetrant disease. *Currently unable to confirm phase. Genomics England did not permit publication of more detailed phenotype data due to confidentiality concerns.

### Assessment of pathogenicity of variants

All SNVs and indels had allele frequencies less than or equal to 4 × 10^−3^ according to gnomAD v3.0 and CADD 1.6 PHRED scores between 15 and 47 (see Table 2). Assessment of pathogenicity of variants was undertaken following the American College of Medical Genetics and Genomics (ACMG) and the Association for Molecular Pathology guidelines for the interpretation of sequence variants^42^. Phasing of GEL whole genome sequencing is not available, and confirmation in participants with possible compound heterozygous mutations required analysis of parent genomes where available. However, the compound heterozygous missense mutations must be *in-trans* with heterozygous CNV deletions, whereas homozygous variants do not require phase information. Assessment of parental genomes for segregation of candidate variants was possible in three cases. Participant nine was possibly compound heterozygous for R668C and S666C; however, both variants were present in the father, confirming presence *in-cis*. Participant thirty-three was possibly compound heterozygous for R545K and R444C; however, both variants were present in the participant's mother, confirming presence *in-cis*. Compound heterozygous variants in participant fourteen (R639I and Y78H) were confirmed to segregate with one variant in each parent. No variants were demonstrated to be de novo. No variants were predicted to affect splicing, including many intronic variants with moderate to high CADD scores. A list of these variants, including heterozygous pathogenic variants and those that failed to segregate or were predicted benign in ClinVar, are included in supplemental data (see Supplementary Table 1).

**Table 2:**
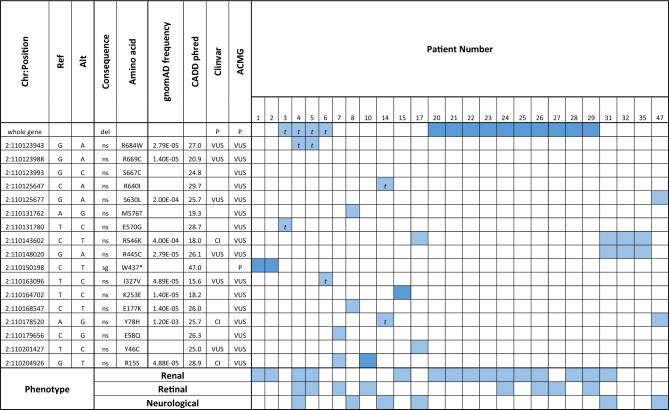
Eighteen pathogenic or assumed pathogenic variants from 26 individuals consistent with autosomal recessive NPHP1-related disease.

Dark blue background indicates homozygous variants, and pale blue indicates heterozygous variants; *t* in-trans. *Chr:Position* chromosome and position of 5′ base of variant in *Homo sapiens* (human) genome assembly GRCh38, *Ref* reference allele, *Alt* alternative allele, *Consequence* Consequence of the variant (*del* CNV whole gene deletion of NPHP1, *sg* stop gained variant, *ns* non-synonymous), *Amino acid* amino acid change, *gnomAD frequency* gnomAD genome v3.0 allele frequency (all populations), *CADD phred* Combined Annotation Dependent Depletion score (phred scale) version 1.6, *Clinvar* Clinical significance according to Clinvar (*P* pathogenic, *VUS* variant of uncertain significance, *CI* conflicting interpretations of pathogenicity), *ACMG* assessment of pathogenicity after application of American College of Medical Genetics and Genomics and the Association for Molecular Pathology guidelines for the interpretation of sequence variant (*P* pathogenic, *VUS* variant of uncertain significance).

### Copy number variant (CNV) analyses

CNV analysis revealed 424/78,050 participants from 344 families (0.54%) with CNV deletions identified across cancer and rare disease cohorts. The allele frequency of CNV deletions was similar in cancer and rare disease cohorts and twice the rate described in gnomAD SV v2.1 (see Table 3). The latter may not be surprising given exome-based sequencing is more challenging for calling CNVs.

**Table 3:** Heterozygous and homozygous *NPHP1* CNV deletions and allele frequencies for rare disease, all cancer germline compared with gnomAD v2.1 SV.

	Rare disease participants in aggregated gVCF	Cancer participants with germline DNA in aggregated gVCF	gnomAD v2.1 SV
Heterozygous CNV (total number of individuals)	331	83	39
Homozygous CNV (total number of individuals)	9	1	0
Total allele count	349	85	39
Number of individuals	62,898	15,152	21,694
Allele frequency	5.55 × 10^–3^	5.61 × 10^–3^	1.80 × 10^–3^

Ten homozygous CNV deletions from ten different families were joint ranked twenty in order of descending GenePy score. Six of these were not previously reported by the GEL genomic medicine centres. Eight participants with homozygous CNV deletions had renal failure with either ESKD (n = 6) or CKD stage 4 (n = 2). Age at first recorded renal phenotype using either HES ICD-10 data or recruitment HPO terms ranged from 6 to 52 years. Four participants had retinal dystrophy with age at first recorded retinal phenotype ranging from 25 to 54. One participant had no phenotype data available but had been recruited due to 'unexplained renal failure in the young', which required onset of ESKD before the age of 50. One participant was recruited into the cancer cohort for malignant melanoma and had ESKD with tubulointerstitial nephritis and hypertensive renal disease by age 52 and hereditary retinal dystrophy by age 54. All ten patients with CNV deletions were deemed pathogenic by ACMG guideline standards due to the predicted null variant nature of the gene deletion giving very strong evidence of pathogenicity.

### Single nucleotide variants (SNVs)

Eight participants had a phenotype consistent with renal or retinal disease and homozygous or compound heterozygous SNVs. These included a homozygous stop-gain mutation (two siblings from one family), heterozygous CNV deletions with a missense mutation (two siblings from one family), two different homozygous missense mutations (two individuals from two families), and two different possible compound heterozygous missense mutations (two individuals from two families). The participants with homozygous stop gained and homozygous missense mutations and three of the ten with homozygous CNV deletions were known to have consanguinity. Of these eight the two siblings with homozygous stop-gained variants could be reported as pathogenic according to ACMG guidelines due to the null variant. The other SNVs were rare (4.00E−04) or novel missense variants with strong in-silico evidence of pathogenicity with CADD scores of between 18 and 28.9.

An additional eight participants from eight families had recessive genotypes consistent with monogenic *NPHP1*-related disease but without a documented phenotype. These included six with possible compound heterozygous missense mutations and two compound heterozygous CNV deletions *in-trans* with a missense mutation. One participant without a documented renal or retinal phenotype was recruited to the cancer cohort. The other was the relative of a participant recruited with intellectual disability and no phenotype data available for renal or retinal disease.

Many heterozygous CNV deletions (n = 414) were discovered, including six in the top 50 GenePy rankings. Two heterozygous CNV deletions were identified in participants recruited to the cancer cohort, but no renal or retinal phenotype was recorded. However, the deletions were in-trans with an intronic variant with a CADD 1.6 PHRED score of 15.56 and found in an active enhancer mark (H3K27ac). The remaining heterozygous CNVS were joint ranked 593 and not found with any additional predicted pathogenic variants by CADD 1.6.

### AlphaFold structural modelling of variants

**Figure 2:**
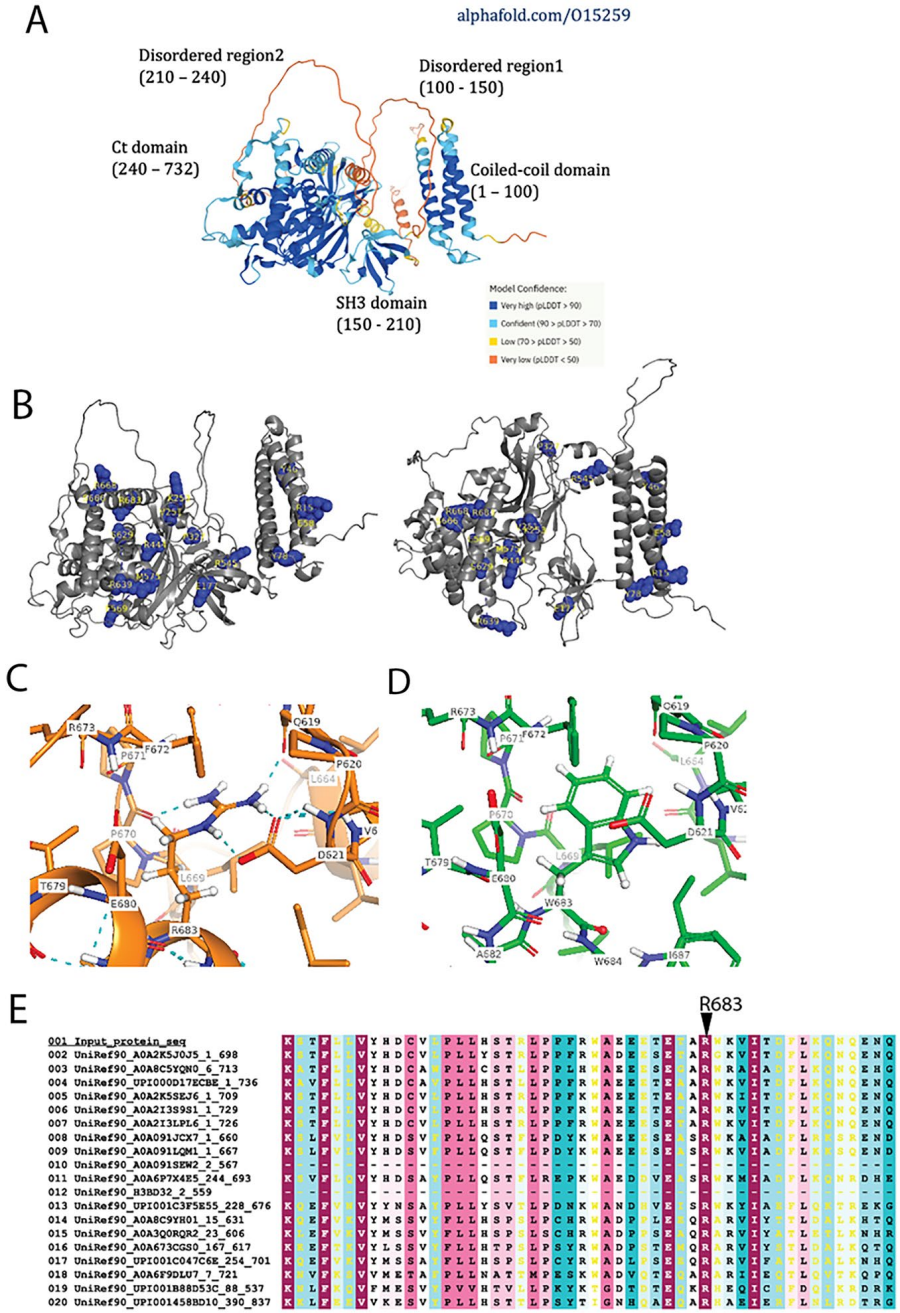
Alphafold modelling of NPHP1 variants. (**A**) Model of the NPHP1 polypeptide as predicted by AlphaFold and manually annotated by the main regions predicted. (**B**) Mapping of mutations identified by GenePy within the NPHP1 structural model. (**C**) Residue R683 and its predicted side-chain entourage in NPHP1 model. (**D**) Residue W683 and its predicted side-chain entourage in NPHP1-R683W mutant model. In (**C**) and (**D**) H-bonds are depicted as cyan dotted lines. (**E**) Multiple sequence alignment showing conservation of R683 in NPHP1 orthologues (mainly vertebrates); the colour coding ranges according to the level of phylogenetic conservation, from variable (cyan) and average (white) to conserved (maroon).

To explore if any the pathogenic or assumed pathogenic variants (described in Table 2) could have an impact in the structural integrity of NPHP1, we mapped the most conspicuous changes into the structural model of NPHP1 (UNIPROT#O15259) recent released by AlphaFold^43,44^. In short, the AlphaFold algorithm is an AI system based on the analysis of contact maps of co-evolving residues extracted from multiple sequence alignments (MSAs) of sequences, which feed two neural networks: one trained on Protein Data Bank (PDB) structures to predict interatomic angles and distances; another, trained to score the geometry and structural accuracy. This model identifies five different structural regions within the NPHP1 polypeptide chain (see Fig. 2): an N-terminal domain enriched by coil-coiled helical structures (residues 1 to 100, approximately), a first disordered region 1 (residues 100 to 150), a SH3 domain (r.150 to 210), a second disordered region (210 to 240), and a globular C-terminal domain rich in both beta-sheets and alpha helices (240 to 732). The quality of the prediction in generally consistently high in most of these regions, with the exclusion the two disordered regions. Experimental structural data is available for the N-terminal coil-coiled domain, like that of the BAG domain protein family, known to mediate the anti-apoptotic functions, and the SH3 domains thought to be involved in cell adhesion^45,46^.

The structural model of NPHP1 allowed assessment of the emplacement and possible impact on the structure of the mutations identified using both a missense predictor and Maestro Suite v13.1 and BioLuminate 4.6 Release 2022-1 (Maestro & BioLuminate, Schrödinger, LLC, New York, NY, 2021)^37–41^. Firstly, all the mutations were found located in predicted domain-containing regions (Supplementary Fig. 1B and Supplementary Table 2). Secondly, most mutations were predicted not to have a significant impact on the structure—except for M575T and R683W; however, for M575T a significant change in the cavity is predicted given the residues size reduction (not shown). Also, for R684W on the other side, the prediction on change include: this substitution replaces a buried charged residue with an uncharged residue (TRP), disrupts multiple H-bond with the residues P670, D621, Q619, and disrupts a salt bridge formed by NE atom of R683 and OD1 atom of D621. Along with a contraction of 90.504 Å^3^ (Supplementary Fig. 1C,D; Supplementary Table 3). Finally, we confirmed that R683 was phylogenetically conserved (Supplementary Fig. 1E).

## Discussion

Eighteen participants were identified with genotypes consistent with a diagnosis of recessive *NPHP1*-related disease and renal or retinal phenotypes. Of these, ten participants with homozygous *NPHP1* deletions could be considered pathogenic according to ACMG guidelines. Eight patients had homozygous and compound heterozygous SNVs, including two heterozygous variants of uncertain significance (VUS) in-trans with CNV deletions. Of these, only the two with homozygous stop gain mutations can be classified as pathogenic according to ACMG criteria.

### Evidence for pathogenicity

The other SNVs were rare (4.00E−04) or novel missense variants with strong in-silico evidence of pathogenicity. The CADD PHRED scores were between 18 and 28.9. At the risk of losing some causative variants, we chose a cut off CADD score of 15 and above. This cut-off was chosen as it represents the median value for all possible canonical splice site changes and non-synonymous variants in CADD. Variants with CADD PHRED scores over 15 are predicted to be in the top 0.5% of most deleterious substitutions that can occur in the human genome. However, as is true of missense variants in many other genes they cannot be officially classified as clinically pathogenic without expensive and time consuming in-vitro or in vivo functional assays. This poses a wider problem with the bottle neck to clinical interpretation now moving from high through put genomics to a lack of high throughput functional assays to confirm pathogenicity. Given these variants are rare or even private to a specific family, the cost of developing 'well-established in vitro or in vivo functional studies' is likely to be prohibitive and result in a loss of translational benefit to patients and their families. Where functional experimental evidence is available it supports roles of some protein domains in apoptosis and cell adhesion^45,46^. AlphaFold structural modelling of these SNVs additionally predicted significant impact on protein structure.

### Identification of participants who may yet to develop disease

An additional eight participants had a recessive SNV genotype consistent with monogenic *NPHP1-*related disease but no documented renal or retinal phenotype. One was recruited with cancer, and three were 'unaffected' relatives. Generally, phenotype data are scarce for participants recruited to cancer and for rare disease participants other than the proband. The GEL recruitment process for cancer participants does not routinely require documentation of kidney function, even though this would invariably have been tested in all patients undergoing treatment for their cancer. 'Unaffected' relatives with no phenotype recorded at recruitment and no longitudinal ICD-10 HES data could be due to either not being clinically evaluated, having subclinical disease, or having a clinical disease that is not documented, that may present later or is not fully penetrant.

When assessing compound heterozygosity, the difficulty is that some variants may lie on the same chromosome. This cannot currently be assessed with short-read data such as that used by Genomics England. We confirmed the presence in-trans in one participant through parental assessment. Their young age may explain the lack of phenotype data for probands with compound heterozygous variants. They may yet develop significant disease in their lifetime (two were under 15 years old). Array-based data on homozygous *NPHP1* CNV deletions from Snoek et al*.* suggests that individuals may not reach end-stage kidney disease until the seventh decade^21^. Longitudinal follow up of these participants using HES data from GEL will be required to clarify penetrance and confirm other cases.

### Benefits and limitations of gene first approach using GenePy

The large number of participants in the 100kG project offered a unique opportunity to identify the full spectrum of pathogenic variation in *NPHP1* and related diseases. We therefore applied a more objective, agnostic genotype-to-phenotype approach. A significant benefit over a case–control approach is that it precludes the need to exclude participants from analyses based on predicted ancestry or relatedness. This is particularly important given the under-representation of individuals from non-European ancestries in the genetic literature. As with all approaches that implement short read data, this approach is subject to the same limitations. This includes reduced sensitivity to detect variants in highly variable regions and absence of phase representation. A lack of phase data is particularly problematic in adult-onset disease where parent DNA may not be available to determine zygosity, therefore limiting the ability to assess compound heterozygous variants. As with all large-scale data, GenePy scoring is dependent upon data quality and the elimination of systematic bias and technical artefacts. However, the genomics England data used was of high integrity, with all samples sequenced with 150 bp paired-end reads in a single lane of an Illumina HiSeq X.

### Potential for diagnostic uplift

Our data support the benefit of a gene first approach that allows the identification of genotypes and associated phenotypes unbiased with respect to their phenotype. This approach can be applied to any gene and includes participants who may yet to develop disease. Notably, the genotype–phenotype approach did not miss any individuals reported as solved by the GEL clinical interpretation pipeline at the time of writing. According to GMC exit questionnaire data, nine participants from seven families were reported by GEL using the clinical interpretation pipeline. However, one was reported for another retinal gene (*PDE6B*) other than *NPHP1*. This highlights the possibility that some participants have more than one monogenic molecular diagnosis. In one whole exome study of 7374 patients, 101 (4.9%) had diagnoses that involved more than one locus, and associated phenotypes could be distinct or overlapping^47^. All patients with more than one genetic diagnosis were known to be consanguineous.

### The contribution of SNVs to *NPHP1*-related disease

Homozygous CNV deletions of *NPHP1* are a well-defined cause of kidney failure. However, the contribution of SNVs and indels to kidney and retinal disease is less well characterised. This study suggests that pathogenic *NPHP1* variants are more common than previously estimated and that SNVs may account for up to 44% of diagnoses of *NPHP1*-related monogenic disease. This may represent a conservative estimate as participants with no recorded renal or retinal phenotype (due to young age or lack of identification) may yet demonstrate these phenotypes.

## Conclusion

The gene first (genotype-to-phenotype) approach combined with a gene pathogenicity score (GenePy) enabled the identification of the full molecular genetic spectrum of nephrocystin-1 (*NPHP1*) in the UK 100,000 genomes project irrespective of relatedness or ancestry. Supportive data from AlphaFold structural modelling suggests that up to 44% of diagnoses of *NPHP1*-related disease may be caused by SNVs in the addition to the well described CNV deletions. Increasing availability of phased genomic data from long read sequencing is expected to further the power of this approach and allow confident assessment of compound heterozygous variants. Longitudinal follow up will provide data on the ability to predict future disease states in high GenePy scoring individuals.

## Supplementary Information

Supplementary Information.

## Data Availability

Full data are available in the Genomic England Secure Research Environment. Access is controlled to protect the privacy and confidentiality of participants in the Genomics England 100,000 Genomes Project and to comply with the consent given by participants for use of their healthcare and genomic data. Access to full data is permitted to researchers after registration with a Genomics England Clinical Interpretation Partnership (GeCIP) (https://www.genomicsengland.co.uk/about-gecip/for-gecip-members/data-and-data-access/) and by contacting the corresponding author upon reasonable request.
