# Supplementary material for: A genotype-to-phenotype approach suggests under-reporting of single nucleotide variants in nephrocystin-1 (NPHP1) related disease (UK 100,000 Genomes Project)
Source: Sci Rep. 2023 Jun 9;13:9369. doi: 10.1038/s41598-023-32169-4 (PMC10256716; doi:10.1038/s41598-023-32169-4)
Supplement: Supplementary file 1 — Supplementary Information. [file 41598_2023_32169_MOESM1_ESM.pdf]

# A genotype-to-phenotype approach suggests under-reporting of single nucleotide variants in Nephrocystin-1 (*NPHP1*) related disease (UK 100,000 Genomes Project).

Gary Leggatt*[1,3], Guo Cheng[1], Sumit Narain[1], Luis Briseño-Roa[2], Jean-Philippe Annereau[2], Genomics England Research Consortium, Christine Gast[1,3], Rodney D Gilbert[1,4], Sarah Ennis[1].

1 - University of Southampton, United Kingdom

2 - Imagine Institute for Genetic Diseases, France

3 - Portsmouth Hospitals NHS Trust, United Kingdom

4 - Southampton Children's Hospital, United Kingdom

# SUPPLEMENTARY INFORMATION

**Supplementary Table 1 - Twenty six additional variants in *NPHP1* from twenty four participants that are not overtly consistent with autosomal recessive inheritance or are recessive with intronic variants or otherwise predicted benign.** Dark blue background indicates homozygous variants and pale blue indicates heterozygous variants; t = in-trans, c = in-cis. Chr:Position, chromosome and position of 5' base of variant in Homo sapiens (human) genome assembly GRCh38; Ref, reference allele; Alt, alternative allele; Consequence, Consequence of the variant (del = deletion, sg = stop gained variant, ns = non-synonymous, int = intronic); Amino acid, amino acid change; gnomAD frequency, gnomAD genome v3.0 allele frequency (all populations); CADD phred, Combined Annotation Dependent Depletion score (phred scale) version 1.6; Clinvar, Clinical significance according to Clinvar (B = benign, P = pathogenic, VUS = variant of uncertain significance, CI = conflicting interpretations of pathogenicity); ACMG, Assessment of pathogenicity after application of American College of Medical Genetics and Genomics and the Association for Molecular Pathology guidelines for the interpretation of sequence variant (P = pathogenic, VUS= variant of uncertain significance).

| Chr:Position | Ref | Alt | Consequence | Amino acid | gnomAD frequency | CADD phred | Clinvar | ACMG | 9 | 11 | 12 | 13 | 16 | 18 | 19 | 30 | 33 | 34 | 36 | 37 | 38 | 39 | 40 | 41 | 42 | 43 | 44 | 45 | 46 | 48 | 49 | 50 |
|---|---|---|---|---|---|---|---|---|---|---|---|---|---|---|---|---|---|---|---|---|---|---|---|---|---|---|---|---|---|---|---|---|
| 2:110123912 | T | G | ns | Q693P | | 24.5 | | VUS | | | | | | | | | | | | | | | | | | | | | | | | |
| 2:110123956 | CTCT | C | del | EE678E | 1.40E-05 | 18.5 | | VUS | | | | | | | | | | | | | | | | | | | | | | | | |
| 2:110123988 | G | A | ns | R668C | 1.40E-05 | 20.9 | VUS | VUS | *c* | | | | | | | | | | | | | | | | | | | | | | | |
| 2:110123993 | G | C | ns | S666C | | 24.8 | | VUS | *c* | | | | | | | | | | | | | | | | | | | | | | | |
| 2:110124045 | T | G | ns | K649Q | | 26.4 | | VUS | | | | | | | | | | | | | | | | | | | | | | | | |
| 2:110131733 | G | A | sg | R585* | 2.10E-05 | 41 | P | P | | | | | | | | | | | | | | | | | | | | | | | | |
| 2:110134860 | C | T | int | | | 15.5 | | VUS | | | | | | | | | | | | | | | | | | | | | | | | |
| 2:110135250 | A | C | int | | 4.54E-02 | 15.96 | | VUS | | | | | | | | | | | | | | | | | | | | | | | | |
| 2:110135250 | A | C | int | | 4.54E-02 | 15.96 | | VUS | | | | | | | | | | | | | | | | | | | | | | | | |
| 2:110143602 | C | T | ns | R545K | 4.00E-04 | 18.03 | CI | VUS | | | | | | | | | *c* | *c* | | | | | | | | | | | | | | |
| 2:110144463 | GGAAA | G | int | | | 17.21 | | VUS | | | | | | | | | | | | | | | | | | | | | | | | |
| 2:110144470 | A | C | int | | | 15.94 | | VUS | | | | | | | | | | | | | | | | | | | | | | | | |
| 2:110148020 | G | A | ns | R444C | 2.79E-05 | 26.1 | VUS | VUS | | | | | | | | | *c* | *c* | | | | | | | | | | | | | | |
| 2:110150028 | T | A | int | | 4.89E-05 | 15.87 | | VUS | | | | | | | | | | | | | | | | | | | | | | | | |
| 2:110150237 | G | A | ns | T423I | | 31 | | VUS | | | | | | | | | | | | | | | | | | | | | | | | |
| 2:110161686 | G | A | sg | R346* | | 43 | P | P | | | | | | | | | | | | | | | | | | | | | | | | |
| 2:110161982 | T | G | int | | 1.11E-02 | 15.56 | | VUS | | | | | | | | | | | | | | | | | | | | | | | | |
| 2:110163071 | G | A | ns | T334M | 5.00E-04 | 15.85 | B | B | | | | | | | | | | | | | | | | | | | | | | | | |
| 2:110168459 | T | A | ns | Y206F | 6.99E-06 | 23.6 | | VUS | | | | | | | | | | | | | | | | | | | | | | | | |
| 2:110168504 | A | T | ns | I191K | | 22.2 | | VUS | | | | | | | | | | | | | | | | | | | | | | | | |
| 2:110169913 | C | A | sg | E139* | 6.98E-06 | 35 | P | P | | | | | | | | | | | | | | | | | | | | | | | | |
| 2:110178469 | G | A | sg | Q95* | | 37 | | P | | | | | | | | | | | | | | | | | | | | | | | | |
| 2:110179677 | G | A | sg | Q51* | 7.00E-06 | 40 | | P | | | | | | | | | | | | | | | | | | | | | | | | |
| 2:110182950 | A | G | int | | | 15.97 | | VUS | | | | | | | | | | | | | | | | | | | | | | | | |
| 2:110200851 | T | C | int | | 2.20E-03 | 15.92 | | VUS | | | | | | | | | | | | | | | | | | | | | | | | |

**Supplementary Table 2. Predicted interactions found in NPHP1 wild-type and R683W models using BioLuminate.**

| Residues | Distance(A) | Specific Interactions | #HB | #Salt Bridges | #Pi Stacking | #Disulfides | #vdW Clash |
|---|---|---|---|---|---|---|---|
| **R683 (wild-type, A:Arg 683)** | | | | | | | |
| A:Ala 682 | 1.3 | | 0 | 0 | 0 | 0 | 0 |
| A:Val 686 | 2.7 | | 0 | 0 | 0 | 0 | 0 |
| A:Thr 681 | 3.3 | | 0 | 0 | 0 | 0 | 0 |
| A:Lys 685 | 3.2 | | 0 | 0 | 0 | 0 | 0 |
| A:Trp 684 | 1.3 | | 0 | 0 | 0 | 0 | 0 |
| A:Pro 670 | 1.9 | 1x hb to A:Pro 670 | 1 | 0 | 0 | 0 | 0 |
| A:Glu 680 | 2.7 | | 0 | 0 | 0 | 0 | 0 |
| A:Thr 679 | 2.0 | 1x hb to A:Thr 679 | 1 | 0 | 0 | 0 | 0 |
| A:Leu 669 | 2.2 | | 0 | 0 | 0 | 0 | 0 |
| A:Ile 687 | 2.0 | 1x hb to A:Ile 687 | 1 | 0 | 0 | 0 | 0 |
| A:Asp 621 | 2.0 | 2x hb, 1x salt bridge to A:Asp 621 | 2 | 1 | 0 | 0 | 0 |
| A:Phe 672 | 2.3 | | 0 | 0 | 0 | 0 | 0 |
| A:Gln 619 | 2.0 | 1x hb to A:Gln 619 | 1 | 0 | 0 | 0 | 0 |
| A:Pro 671 | 3.0 | | 0 | 0 | 0 | 0 | 0 |
| A:Val 622 | 2.4 | | 0 | 0 | 0 | 0 | 0 |
| A:Arg 673 | 3.8 | | 0 | 0 | 0 | 0 | 0 |
| **R683W (A:Trp 683)** | | | | | | | |
| A:Ala 682 | 1.3 | | 0 | 0 | 0 | 0 | 0 |
| A:Thr 681 | 3.2 | | 0 | 0 | 0 | 0 | 0 |
| A:Lys 685 | 3.1 | | 0 | 0 | 0 | 0 | 0 |
| A:Trp 684 | 1.3 | | 0 | 0 | 0 | 0 | 0 |
| A:Pro 670 | 2.4 | | 0 | 0 | 0 | 0 | 0 |
| A:Glu 680 | 2.6 | | 0 | 0 | 0 | 0 | 0 |
| A:Thr 679 | 1.8 | 1x hb to A:Thr 679 | 1 | 0 | 0 | 0 | 0 |
| A:Val 686 | 2.6 | | 0 | 0 | 0 | 0 | 0 |
| A:Leu 669 | 2.3 | | 0 | 0 | 0 | 0 | 0 |
| A:Ile 687 | 1.7 | 1x hb, 1x clash to A:Ile 687 | 1 | 0 | 0 | 0 | 1 |
| A:Asp 621 | 2.6 | | 0 | 0 | 0 | 0 | 0 |
| A:Val 622 | 2.0 | | 0 | 0 | 0 | 0 | 0 |
| A:Phe 672 | 2.4 | | 0 | 0 | 0 | 0 | 0 |
| A:Pro 671 | 2.4 | | 0 | 0 | 0 | 0 | 0 |
| A:Gln 619 | 2.4 | | 0 | 0 | 0 | 0 | 0 |

**Supplementary Table 3. Distribution on missense mutation on the NPHP1 protein model, and their predicted effect in the structure.**

| Mutation | No Structural Damage | Buried Charge Introduced | Buried Charge Replaced | Buried Glycine Replaced | Buried Salt Bridge Breakage | Glycine in a Bend | Buried H-Bond Breakage | Cavity Altered | Domain |
|---|---|---|---|---|---|---|---|---|---|
| **R683W** | | | buried charged residue with an uncharged residue | | NE atom of ARG 683 and OD1 atom of ASP 621 | | Disruption on H-bonds side chains | Contraction of cavity volume >90 Å^3. | C-terminal domain |
| **R668C** | x | | | | | | | | |
| **S666C** | x | | | | | | | | |
| **R639I** | x | | | | | | | | |
| **S629L** | x | | | | | | | | |
| **M575T** | | | | | | | | Contraction of volume by >100 Å^3. | |
| **E569G** | x | | | | | | | | |
| **R545K** | x | | | | | | | | |
| **R444C** | x | | | | | | | | |
| **W436*** | x | | | | | | | | |
| **I326V** | x | | | | | | | | |
| **K253E** | x | | | | | | | | |
| **E177K** | x | | | | | | | | SH3 DOMAIN |
| **Y78H** | x | | | | | | | | COILED-COIL |
| **E58Q** | x | | | | | | | | |
| **Y46C** | x | | | | | | | | |
| **R15S** | x | | | | | | | | |
